# Enabling interoperability across disparate health data sources using Large Language Models

**DOI:** 10.1371/journal.pdig.0001511

**Published:** 2026-07-23

**Authors:** Obinwa Ozonze, Ogechukwu Okonor, Uzondu Dike, Taiwo Adedeji

**Affiliations:** 1 School of Computing, University of Portsmouth, Portsmouth, Hampshire, United Kingdom; 2 Department of Computing & Emerging Technologies, Ravensbourne University London, London, United Kingdom; 3 Insight and Analytics, Emcor Group, Eastleigh, Hampshire, United Kingdom; UMass Lowell: University of Massachusetts Lowell, UNITED STATES OF AMERICA

## Abstract

The heterogeneity of health data systems remains a major barrier to interoperability and data integration, and a challenge for clinical decision support and AI-driven research. The coexistence of multiple Common Data Models (CDMs) complicates integration, requiring complex manual or rule-based schema mapping, which is labor-intensive, error-prone, and difficult to scale. We evaluated three leading Large Language Models (LLMs): ChatGPT, Gemini, and DeepSeek, for their ability to match data elements from four widely used CDMs (PCORnet, OMOP, Sentinel, and i2b2) to FHIR R4 via web-based chat interfaces. Reference mappings from the HL7 Common Data Models Harmonization Implementation Guide were used as the benchmark. Standardized prompts were submitted across 36 total runs (3 LLMs × 4 schemas × 3 repetitions), and accuracy was measured using F1-scores with 95% confidence intervals, alongside strict (AND) and relaxed (OR) match criteria. DeepSeek obtained the highest numerical mean F1-scores (0.78-0.99) with strong consistency (AND Match = 0.87). Gemini showed good coverage (OR Match = 0.96) but greater variability (AND Match = 0.64), while ChatGPT achieved moderate accuracy with lower consistency (AND Match = 0.51). A two-way ANOVA indicated no statistically significant differences by LLM type (p = 0.138), CDM schema (p = 0.194), or their interaction (p = 0.451). Performance varied by domain, with Demographics and Condition mapped most accurately, while Observation showed the highest error rates (44% of total errors). Out of 2,583 total matchings, 314 errors were observed and categorized into four failure patterns: semantic confusion (42%), structural misalignment (28%), ambiguous terminology (18%), and terminology variance (12%). This study demonstrates the feasibility of using LLMs to automate health data integration as a complementary approach to traditional ontology-based methods, particularly for well-structured, context-rich CDMs. However, safe deployment requires human-in-the-loop validation, governance, prompt optimization, metadata integration, and validation to ensure clinical and research reliability.

## Author summary

Integrating health data from multiple sources can be a laborious task due to incompatible data models, which pose interoperability issues. This study evaluated the feasibility of using large language models (LLMs) to automate schema mapping across four widely used common data models (OMOP, PCORnet, Sentinel, and i2b2) to the FHIR standard. Three leading LLMs (ChatGPT, Gemini, and DeepSeek) were tested via web-based chat interfaces using standardized prompts across 36 total runs (3 LLMs × 4 schemas × 3 repetitions). The results varied across clinical domains and data models. While one model achieved the highest mean F1-scores with strong consistency, a two-way ANOVA found no statistically significant differences across LLMs or CDMs, indicating that the primary contribution lies in demonstrating the feasibility of LLM-based mapping as a class of methods rather than identifying a single superior model. Out of 2,583 total matchings, 314 errors were categorized into four systematic error patterns: semantic confusion between related concepts, structural misalignment in hierarchical mappings, ambiguous terminology requiring additional context, and terminology variance across naming conventions. Our findings suggest that LLMs can reduce the burden of manual schema mapping and offer a scalable alternative to traditional rule- or ontology-based techniques such as WordNet, UMLS, and AgreementMakerLight. However, deployment in clinical or regulatory contexts requires human-in-the-loop validation, governance frameworks to address potential clinical harms from incorrect mappings, and further work on API-based automation to improve reproducibility. This work demonstrates the potential of LLMs as a complementary tool to existing ontology alignment methods, warranting further development and validation in real-world settings.

## Introduction

### Background

Health data contain information about the health and well-being of individuals or populations and exists in various digitized forms that support the capture, organization, and storage of health information. These include research repositories containing clinical trial records and health surveys, patient and disease registries, wearable devices, gene sequencing, and electronic health record systems (EHRs) that store administrative and clinical information from routine healthcare visits [1,2].

There is significant interest in using health data to improve decision making in clinical practice, and to develop artificial intelligence (AI) and machine learning models that can accelerate medical research and deepen our understanding of diseases and treatments [3,4]. However, health data repositories typically use different data structures (schemata) and physical systems (databases) to store and organize data, which creates significant challenges for data integration and interoperability, particularly when data are sourced from multiple systems or used in a distributed environment [5–7].

Addressing this data heterogeneity often involves the use of a common data model (CDM), a generic collection of data elements designed to enable the uniform collection, sharing, organization, storage, querying, and display of data across multiple and disparate information systems and components [8,9]. Examples of established CDMs that various organizations have adopted as standards for aggregating or exchanging health data include the Observational Medical Outcomes Partnership (OMOP), the Fast Healthcare Interoperability Resources (FHIR), Sentinel CDM (SCDM), Informatics for Integrating Biology & the Bedside (i2b2), the Patient-Centered Outcomes Research Network (PCORnet), and Digital Imaging and Communications in Medicine (DICOM) [9–12].

The expectation of adopting CDMs is that when both the source and target data have the same model, data integration will be more streamlined and efficient. However, in practice, this scenario is often challenging to attain due to the existence of multiple CDMs with differing scopes that do not fully capture the specificity and breadth of available health datasets, as well as the use of different naming conventions to encode the semantic meaning and content of data elements. Other barriers, such as licensing fees, limited user community size, and poor maintenance, also contribute to the proliferation of CDMs [13,14]. Some health data repositories also rely on locally defined data element sets and bespoke data models, and do not utilize any of the established CDMs.

### Schema mapping

When source and target data models do not match, more complex data integration and normalization processes are required to consolidate data from the different sources into a unified model or view. These processes often include Extract, Transform, and Load (ETL) operations, which typically rely on schema mapping of source and target data elements. Such mappings are often hardcoded or defined manually using interactive interfaces or configuration files [15]. However, manually defining schema mappings can be labor-intensive, require extensive domain knowledge, and be prone to errors, particularly in large-scale and dynamic environments [16–18].

To address these challenges, automating schema mapping has been the focus of extensive research across many application domains, and numerous techniques have been proposed, many of which rely on data element names to identify similar attribute pairs between schemas [19–23]. These techniques search for exact term matches, or syntactical similarities, while more advanced approaches explore semantic similarity by leveraging dictionaries and lexical databases, such as WordNet [22,24,25] to determine word meanings and match attributes. The latter is based on the premise that data element names consist of semantically related words whose meanings are interrelated.

According to ISO 11179, a data element name may include an object term representing the domain described by the property term, a qualifier term that distinguishes it from others, and a representation term indicating the type of data stored. These terms are typically organized in the following sequence: < object> <qualifier> <property> <representation> [26,27]. Hence, using hierarchical network of semantically related words, prior studies have enabled the comparison of data element names based on the proximity of individual tokens within the “is-a” hierarchy or other semantic structures.

Despite their widespread use, semantic networks such as WordNet are not comprehensive. Although they provide substantial coverage of general vocabulary, they offer limited support for newly emerging terms and often fail to cover local expressions or non-standard terminology, particularly in specialized domains such as healthcare [28,29]. Moreover, traversing these databases to compute semantic similarity can be computationally intensive, thereby slowing the matching process [30].

### Study objectives

This study aims to evaluate the effectiveness of LLMs in automating schema mapping for health data models using data element names. It investigates whether LLMs can address the limitations of traditional dictionary and ontology-based methods, such as WordNet, in identifying semantically equivalent data elements across heterogeneous schemas. The overarching goal is to develop a scalable, data-driven approach to schema mapping that reduces manual effort, enhances interoperability, and facilitates broader integration of health data.

## Materials and methods

### Large Language Models

Three prominent LLMs, including OpenAI’s ChatGPT [31], Google’s Gemini [32] and DeepSeek [33], were selected to evaluate the effectiveness of LLMs in automating schema mapping across diverse health data models.

ChatGPT is a transformer-based LLM model optimized for generating human-like responses to natural language prompts on a wide range of topics [34]. Gemini employs a multimodal architecture capable of processing text, images, audio and video [32,35]. DeepSeek uses a mixture-of-experts (MoE) framework to optimize computational efficiency while maintaining strong generative performance [33,36].

Although these LLMs differ in architecture, they share the common feature of producing semantically meaningful outputs from complex, heterogeneous data, including text. In this study, the LLMs were evaluated for their ability to automate exact and semantically related data element name matching, including names that contain synonyms, abbreviations, alphanumeric terms, or typographical errors.

### Data models and preparation

The evaluation employed five widely used health data models, each representing a distinct schema structure: PCORnet CDM v4, OMOP v5.2, Sentinel v6.0.2, i2b2 v1.4, and FHIR R4, and focusing on CDM-to-FHIR transformation, a common clinical data harmonization scenario where legacy research CDMs are integrated into standardized interoperability frameworks. Source-to-target mappings were obtained from the FHIR Common Data Models Harmonization Implementation Guide (CDMH IG v1.0.0: STU 1) [37] and matching performance evaluated each LLM’s ability to reproduce the reference FHIR mappings for the four source CDMs (PCORnet, OMOP, Sentinel, and i2b2).

For consistent and relevant comparison, the study was limited to core domains commonly represented in EHR systems and present across all five CDMs, including patient demographics, clinical encounters, conditions, procedures, and observations. Formatting inconsistencies were also resolved, and domain-level prefixes were appended to attributes to ensure unique and context-aware feature representations, especially for attributes such as “id,” “status,” and “date”.

### Data analysis

Using a realistic workflow evaluation, the mapping tasks were conducted using the web-based chat interfaces of each LLM, including OpenAI ChatGPT (https://chat.openai.com), Google Gemini (https://gemini.google.com), and DeepSeek (https://chat.deepseek.com), between January and April 2025. For each CDM-to-FHIR mapping task, a new chat session was initiated, and a standardized prompt was submitted. The prompt consisted of (1) a list of the source CDM data elements, (2) a list of the target FHIR data elements, and (3) structured instructions specifying the mapping activity and output format. Follow-up prompting was not permitted to ensure experimental consistency and prevent information leakage.

The prompt used was as follows: “*I have provided two data model elements: source and target. Map the source to the target element, where each target element must be matched to one source element. Show all results in one table with headings: SN, target element, source element, and similarity score (ranges from 0 to 1). Only use the attributes provided.*”

To minimize memory effects and prompt history bias, and account for LLM output variability, the same prompt was executed three times for each model-schema pair, resulting in 36 total runs (3 LLMs × 4 schemas × 3 repetitions). Source CDM elements were also randomized across runs to reduce order effects. All outputs were exported to a standardized Excel template. See Supporting information (S1 Data) for the prompt, CDM elements and all raw outputs.

LLM outputs were evaluated by comparing predicted mappings against curated ground-truth mappings to derive the F1-score [38], which was used as the primary evaluation metric. Mapping outputs were also aggregated across repeated runs for each LLM-CDM pair using strict (AND) and relaxed (OR) criteria to assess output stability and mapping coverage. Under the strict criterion, a mapping was considered correct if the predicted FHIR element matched the ground truth across all three runs. Under the relaxed criterion, a mapping was considered correct if the correct element appeared in at least one run. The evaluation also assumed a closed-set mapping scenario, in which each source attribute has at least one valid target in the reference mapping. Where multiple valid targets are present, a prediction is considered correct if any valid target was returned.

## Results

### CDM-FHIR Structure

Table 1 summarizes the structural characteristics of the CDM-to-FHIR reference mapping datasets. Attribute counts varied across CDMs, ranging from 39 attributes in i2b2 to 96 attributes in PCORnet. Most mappings follow a one-to-one correspondence, with only OMOP containing a single one-to-many mapping case. Source-to-target attribute ratios were also greater than one across all CDMs, indicating that multiple source attributes are often mapped to a single FHIR element. These results highlight the structural heterogeneity between FHIR and other CDMs, as well as the complexity of clinical data integration within standardized interoperability frameworks.

**Table 1:** Analysis of mapping relationships in the ground-truth CDM-FHIR dataset.

Source CDM	No. source attributes	No. target attributes (FHIR)	1-to-1 mappings	1-to-many mappings	Source-to-target ratio
PCORnet	96	70	96	0	1.37
OMOP	86	56	85	1	1.54
Sentinel	66	52	66	0	1.27
i2b2	39	25	39	0	1.56

### Overall LLM Performance

Mapping performance across the three evaluated LLMs: ChatGPT, Gemini, and DeepSeek, is presented in Table 2. In this sample, DeepSeek obtained the highest numerical mean F1-scores across the CDM-FHIR tasks, ranging from 0.78 to 0.99. -Performance was relatively stable across repeated inference runs, particularly for PCORnet-to-FHIR and i2b2-to-FHIR mappings, as indicated by the narrow confidence intervals. Gemini showed good performance on PCORnet and Sentinel mappings (mean F1 = 0.90) but exhibited higher variability on the i2b2 mapping task than other LLMs. ChatGPT achieved moderate F1-scores overall, with lower performance for OMOP and Sentinel mappings (0.64 and 0.63, respectively), and greater variability across CDMs.

**Table 2:** Mapping performance of LLMs across health data models.

LLM	Task	AND Match	OR Match	Mean F1 Score	95% CI
ChatGPT	PCORnet vs. FHIR	0.61	0.99	0.82 ± 0.09	[0.61–1.00]
	OMOP vs. FHIR	0.34	0.88	0.64 ± 0.19	[0.17–1.00]
	Sentinel vs. FHIR	0.23	0.90	0.63 ± 0.34	[0.00–1.00]
	i2b2 vs. FHIR	0.84	1.00	0.93 ± 0.06	[0.78–1.00]
Gemini	PCORnet vs. FHIR	0.81	0.96	0.90 ± 0.04	[0.82–0.99]
	OMOP vs. FHIR	0.66	0.91	0.79 ± 0.03	[0.73–0.86]
	Sentinel vs. FHIR	0.83	0.96	0.90 ± 0.02	[0.85–0.95]
	i2b2 vs. FHIR	0.24	1.00	0.75 ± 0.42	[0.00–1.00]
DeepSeek	PCORnet vs. FHIR	0.94	0.97	0.96 ± 0.02	[0.92–1.00]
	OMOP vs. FHIR	0.73	0.82	0.78 ± 0.03	[0.71–0.86]
	Sentinel vs. FHIR	0.85	0.92	0.87 ± 0.01	[0.86–0.89]
	i2b2 vs. FHIR	0.96	1.00	0.99 ± 0.02	[0.93–1.00]

Across all CDMs, OR matching scores were consistently high for all LLMs, typically exceeding 0.90, and i2b2-to-FHIR mappings achieved perfect coverage (OR = 1.00) across all LLMs, indicating that all LLMs were generally able to identify valid schema correspondences after three attempts. In contrast, the AND matching scores were more variable, particularly for OMOP and Sentinel mappings with ChatGPT, reflecting reduced consistency in identifying complete correspondences in these tasks.

### Performance across CDMs

As shown in Table 3, the evaluated LLMs demonstrated generally good schema matching capabilities across diverse CDMs. PCORnet and i2b2 achieved the highest mean F1-scores (≈0.89), followed by Sentinel (0.80), with OMOP performing lowest (0.74).

**Table 3:** Mean F1 scores of LLMs across CDM-FHIR mapping tasks.

CDM/ LLM	ChatGPT	Gemini	DeepSeek	Average
PCORnet	0.82	0.90	0.96	0.89
OMOP	0.64	0.79	0.78	0.74
Sentinel	0.63	0.90	0.87	0.80
i2b2	0.93	0.75	0.99	0.89

Analysis of CDM structural features revealed no consistent linear relationship with mapping performance. For instance, i2b2, with a high source-to-target attribute ratio (1.56), achieved strong performance (0.89), whereas OMOP, with a comparable ratio (1.54), showed lower performance (0.74). Similarly, data element verbosity, measured as the average number of terms and character length (Table 4), did not directly predict mapping success. FHIR and OMOP had longer and more detailed attribute names, yet OMOP showed lower performance (mean F1 ≈ 0.74), whereas i2b2 and PCORnet, with moderate verbosity, achieved higher F1-scores. Sentinel, with shorter names, also had relatively lower performance.

**Table 4:** Average number of terms and character length per data element in PCORnet, OMOP, Sentinel, i2b2, and FHIR.

CDM	Avg. number of terms	Avg. character length
PCORnet	3.25	18.70
OMOP	4.52	29.63
Sentinel	2.83	19.40
i2b2	3.97	26.03
FHIR	5.14	35.30

A two-way ANOVA with replication further indicated that LLM type (p = 0.138), CDM schema (p = 0.194), and their interaction (p = 0.451) were not statistically significant. While these statistical findings should be interpreted cautiously due to the limited number of runs per condition (n = 3), they suggest that no LLM consistently outperformed others across CDMs, and that CDM structural complexity did not systematically favor or disadvantage any LLM. Nonetheless, the data element verbosity trends, though exploratory, highlight the potential importance of element verbosity, suggesting that moderate verbosity may provide sufficient contextual information for mapping, whereas overly verbose or highly generic terms could increase semantic complexity and reduce consistency.

### Matching error analysis

To further contextualize these findings, we analyzed mapping errors across the core clinical domains (Table 5). Out of 2,583 total matchings, 314 errors were observed, with Observation elements being the most challenging to match, accounting for approximately 44% of total errors, followed by Procedures (18.5%) and Encounters (15.9%). Conditions (13.4%) and Demographics (8.3%) were relatively easier to map. Across LLMs, DeepSeek consistently produced the fewest errors, followed by Gemini, while ChatGPT produced the highest number of errors. Table 6 lists the top 10 data elements with the highest number of mapping errors, many of which occur in the Procedure and Observation domains.

**Table 5:** Error rates across five core clinical domains.

Domain	ChatGPT	Gemini	DeepSeek	Total	%Total Errors
Condition	20	13	9	42	13.4
Encounter	30	13	7	50	15.9
Observation	67	38	33	138	43.9
Demographics	14	9	3	26	8.3
Procedure	30	15	13	58	18.5
Total	161	88	65	314	100

**Table 6:** Top 10 data elements with the highest number of mapping errors.

Source data elements	Error count	Domain	CDM
procedure_occurrence.procedure_date	14	Procedure	OMOP
laboratory result.facility_code	14	Observation	Sentinel
procedure_occurrence.provider_id	13	Procedure	OMOP
procedure.px	13	Procedure	PCORnet
procedure.encounterid	10	Procedure	PCORnet
condition_occurrence.condition_source_value	9	Condition	OMOP
person.birth_datetime	9	Demographics	OMOP
device_exposure.unique_device_id	9	Procedure	OMOP
condition_occurence.condition_type_concept_id	8	Condition	OMOP
encounter.patid	8	Encounter	PCORnet

Analysis of the discrepancies also revealed recurring error patterns (Table 7), which we have grouped into four categories: misalignment of semantically related elements; incorrect mapping due to differences in schema hierarchy or nesting; generic terms lacking context; and inconsistent naming of equivalent concepts across schemas. These patterns suggest that mapping performance may be influenced by domain-specific factors, such as the variability of clinical terminology, hierarchical complexity, and attribute heterogeneity. Observation elements, in particular, appear prone to semantic ambiguity and structural complexity because they span diverse clinical data types, including vital signs (e.g., height, weight, oxygen saturation), laboratory results, and free-text observations. These data are often stored heterogeneously, with varying field structures, classification systems, levels of granularity, and site-specific customizations. Similarly, Procedure-related errors reflect the complexity of event-based and relational attributes. Together, these results indicate that LLM-based schema matching is sensitive to both semantic nuance and structural variation, with recurring error patterns helping to explain differences in performance across domains.

**Table 7:** Error patterns and examples.

Error pattern	Example
Misalignment of semantically related but conceptually different elements.	“procedure_date” maps to “procedure.performedperiod.high” instead of “procedure.performeddatetime”
Incorrect mapping due to differences in schema hierarchy or nesting.	“procedure_occurrence.provider_id” mapped to “procedure.identifier” instead of low-level “procedure.performer.actor.identifier.”
Generic terms lacking context, leading to multiple interpretations.	Field “code” could indicate condition code, encounter type, or procedure code.
Inconsistent naming of equivalent concepts across schemas.	“person.birth_datetime”, “patient.extension: patient-birthtime”, “patient.birthdate” all referring to the same temporal attributes

## Discussions

This study evaluates the feasibility of using LLMs to automate schema mapping across heterogeneous health data models. Without additional training or fine-tuning, we evaluated three prominent LLM models: ChatGPT, Gemini, and DeepSeek, all of which demonstrated promising performance in automating data element mapping across multiple CDMs and clinical domains. These results provide early evidence that LLMs can reduce the manual effort and costs traditionally required for reconciling heterogeneous data structures and streamlining health data interoperability.

Our findings also highlight the potential of LLM-based approaches as a scalable alternative to traditional rule- or ontology-based techniques like WordNet, UMLS, and AgreementMakerLight, which have long been used to automate schema matching [22,25,39,40]. While these traditional approaches provide structured and interpretable solutions, they typically rely on exact term matching, predefined synonyms, or well-curated ontologies, which may limit their flexibility and scalability when handling informal naming conventions, evolving source schemas, or previously unseen data sources. In contrast, LLMs, as observed in this study, enable context-aware reasoning over data element names and can generate semantically coherent mappings without extensive preprocessing or model tuning. In our evaluation, LLMs achieved high accuracy and identified closely related but non-identical elements, including minor naming variations and abbreviations, thereby reducing reliance on separate exact-match models or additional semantic or fuzzy similarity measures. From a cost-benefit perspective, these characteristics translate to lower setup requirements and faster deployment, which is particularly relevant for large-scale or distributed health data initiatives, where manual mapping can be time-consuming and resource-intensive.

Furthermore, our findings highlight several contexts in which LLMs may be applied within the health data integration cycle. For instance, use cases that prioritize reproducibility, such as integrating data generated from wearable devices, IoT sensors, or consumer health applications, where source schemas frequently change or new data partners are onboarded, will benefit from LLMs that produce stable outputs across repeated runs. Our descriptive results suggest some models exhibited narrower run-to-run variability than others, but because the two-way ANOVA did not detect statistically significant differences between LLMs (p = 0.138) and each condition was evaluated in only three runs, we cannot on the basis of this study recommend a specific model as preferable for such deployments; larger-scale benchmarking is needed before any model is positioned as suited to near-real-time integration. Similarly, LLMs demonstrating strong coverage, even with some variability, may be suitable for exploratory data analysis or human-in-the-loop workflows, such as schema discovery and the proposal of initial mappings, which are often faster than manual identification, particularly for large or complex models [41]. It also allows data engineers and researchers to focus on higher-level validation, complex edge cases, and strategic integration decisions.

Nonetheless, to maximize the benefits LLM-assisted schema mapping in ETL (Extract, Transform, Load) pipelines, health information exchanges, or research data aggregation workflows, while mitigating risks, this study recommends a phased strategy for deploying LLMs for health data integration. Initial implementation should focus on high-confidence, low-risk domains, such as patient demographics, which are typically well-structured and standardized, as observed in this study. Once validated, the approach can be extended to more complex domains, including observations, procedures, and heterogeneous clinical measurements, where semantic ambiguity and nested structures pose greater challenges.

This study has several limitations that warrant consideration. First, the CDMs used in our evaluation are commonly involved in FHIR harmonization implementations and, because they may have partial alignment across systems, may not fully capture the variability encountered in real-world healthcare environments, including incomplete or ambiguous metadata and diverse schema representations such as XML or OWL ontologies. Further evaluation is needed to assess performance across more heterogeneous CDMs, real-world clinical datasets, and operational EHR systems to better understand robustness, generalizability, and other practical limitations.

This study also focused on schema-level alignment, a foundational step in health data integration. We employed name-based schema matching for its LLM compatibility and ease of implementation. While effective, this approach struggles when column semantics diverge from naming conventions, as reflected in observed error patterns. Incorporating instance-based matching and additional schema metadata, such as descriptions and value sets, could improve accuracy, though at the cost of larger datasets and higher computational demands. Nonetheless, achieving full interoperability requires more than accurate schema mapping. Semantic alignment at both value-set and concept levels is essential, since structural mapping alone cannot preserve clinical meaning in CDMs that use heterogeneous terminologies such as SNOMED CT, ICD, and LOINC. Building on this, future work can explore extending LLM-based schema mapping to automate value-set crosswalks or integrate with terminology harmonization frameworks to support clinical language understanding and ontology reasoning.

Similarly, while LLMs show promise as scalable, data-driven tools for automated schema mapping, observed failure patterns, including inconsistent handling of nested structures and semantic distinctions, highlight the need for careful validation and a deeper understanding of performance variability, which can help identify LLMs with genuinely superior mapping capabilities. Future work should also explore strategies to enhance LLM consistency and output, including combining multiple LLMs, domain-specific pretraining, prompt optimization, and hybrid approaches that combine LLM outputs with rules, ontologies and terminology services.

Lastly, understanding how LLMs can best be implemented for various health data integration use cases is also critical to maximize their accuracy, reliability, and scalability. For instance, careful attention to AI safety and governance is important, as incorrect mappings could propagate into clinical or research systems, affecting decisions, analyses, or patient safety, particularly in high-stakes domains like Observation. From a regulatory perspective, AI-enabled data integration may also fall under regulatory frameworks such as FDA guidance on software as a medical device and HIPAA mandates. Given the “black box” nature of LLMs, maintaining comprehensive audit trails is critical for tracking mapping decisions and mitigating potential errors or harms. Bias considerations must also be addressed to ensure mappings perform consistently across diverse datasets and care contexts.

## Conclusions

This study demonstrates that LLM-based schema mapping is a feasible and promising approach for health data integration, offering clear benefits in efficiency, reproducibility, and scalability. By leveraging data element names, we show that LLMs can accurately identify semantically equivalent attributes across heterogeneous common data models, thereby reducing the manual effort traditionally required for mapping and supporting interoperability across both structured and semi-structured domains. These results highlight the potential for LLMs to streamline health data workflows and make health data more readily accessible for analysis and decision-making.

We also highlight important challenges, including heterogeneous domains, nested structures, and ambiguous terminology that remain difficult for LLMs to handle consistently. These findings further emphasize the need for hybrid human-AI workflows, robust validation, and governance frameworks to ensure safe and clinically meaningful integration. Incorporating reproducible pipelines, semantic scoring, and transparent evaluation metrics can further enhance reliability, while human oversight remains critical for mitigating risks and validating complex or ambiguous mappings.

By illuminating both the strengths and limitations of LLMs in this context, this work lays the foundation for future research to improve accuracy, extend applicability to more complex and real-world datasets, and integrate semantic-level reasoning. It also paves the way for a future where healthcare data integration is faster, more reliable, and more accessible through the thoughtful application of artificial intelligence

## Supporting information

S1 Data Data preprocessing scripts and standardized prompts used in the study. This ZIP file contains: (1) the exact standardized prompt submitted to all three LLMs across all 36 runs (3 LLMs × 4 schemas × 3 repetitions), (2) the complete set of source-to-target mapping pairs used for preprocessing and standardizing CDM element names across PCORnet, OMOP, Sentinel, and i2b2, and (3) the complete mapping results from the three LLMs for all four Common Data Models (PCORnet, OMOP, Sentinel, i2b2) to FHIR across iterations.
(ZIP)

## References

[1] Jensen PB, Jensen LJ, Brunak S. Mining electronic health records: towards better research applications and clinical care. Nat Rev Genet. 2012;13(6):395–405. doi: 10.1038/nrg3208 22549152

[2] Austin C, Kusumoto F. The application of Big Data in medicine: current implications and future directions. J Interv Card Electrophysiol. 2016;47(1):51–9. doi: 10.1007/s10840-016-0104-y 26814841

[3] Topol E. The Topol review. Preparing the healthcare workforce to deliver the digital future. 2019.

[4] Nordo AH, Levaux HP, Becnel LB, Galvez J, Rao P, Stem K, et al. Use of EHRs data for clinical research: historical progress and current applications. Learn Health Syst. 2019;3(1):e10076. doi: 10.1002/lrh2.10076 31245598 PMC6508843

[5] Weng C. Clinical data quality: a data life cycle perspective. Biostat Epidemiol. 2020;4(1):6–14. doi: 10.1080/24709360.2019.1572344 32258941 PMC7133741

[6] Feldman K, Faust L, Wu X, Huang C, Chawla NV. Beyond volume: the impact of complex healthcare data on the machine learning pipeline. In: Lecture Notes in Computer Science. Springer International Publishing; 2017. p. 150–69. doi: 10.1007/978-3-319-69775-8_9

[7] Wade TD. Traits and types of health data repositories. Health Inf Sci Syst. 2014;2:4. doi: 10.1186/2047-2501-2-4 25825668 PMC4340801

[8] Garza M, Del Fiol G, Tenenbaum J, Walden A, Zozus MN. Evaluating common data models for use with a longitudinal community registry. J Biomed Inform. 2016;64:333–41. doi: 10.1016/j.jbi.2016.10.016 27989817 PMC6810649

[9] Dugas M, Neuhaus P, Meidt A, Doods J, Storck M, Bruland P, et al. Portal of medical data models: information infrastructure for medical research and healthcare. Database (Oxford). 2016;2016:bav121. doi: 10.1093/database/bav121 26868052 PMC4750548

[10] Kapsner LA, Kampf MO, Seuchter SA, Kamdje-Wabo G, Gradinger T, Ganslandt T, et al. Moving towards an EHR data quality framework: the MIRACUM approach. Stud Health Technol Inform. 2019;267:247–53. doi: 10.3233/SHTI190834 31483279

[11] Voss EA, Makadia R, Matcho A, Ma Q, Knoll C, Schuemie M, et al. Feasibility and utility of applications of the common data model to multiple, disparate observational health databases. J Am Med Inform Assoc. 2015;22(3):553–64. doi: 10.1093/jamia/ocu023 25670757 PMC4457111

[12] Ayaz M, Pasha MF, Alzahrani MY, Budiarto R, Stiawan D. The Fast Health Interoperability Resources (FHIR) standard: systematic literature review of implementations, applications, challenges and opportunities. JMIR Med Inform. 2021;9(7):e21929. doi: 10.2196/21929 34328424 PMC8367140

[13] Klann JG, Joss MAH, Embree K, Murphy SN. Data model harmonization for the all of us research program: transforming i2b2 data into the OMOP common data model. PLoS One. 2019;14(2):e0212463. doi: 10.1371/journal.pone.0212463 30779778 PMC6380544

[14] Kent S, Burn E, Dawoud D, Jonsson P, Østby JT, Hughes N, et al. Common problems, common data model solutions: evidence generation for health technology assessment. Pharmacoeconomics. 2021;39(3):275–85. doi: 10.1007/s40273-020-00981-9 33336320 PMC7746423

[15] Tripathi A, Waqas A, Venkatesan K, Yilmaz Y, Rasool G. Building flexible, scalable, and machine learning-ready multimodal oncology datasets. Sensors. 2024;24(5):1634. 38475170 10.3390/s24051634 PMC10933897

[16] Kiourtis A, Nifakos S, Mavrogiorgou A, Kyriazis D. Aggregating the syntactic and semantic similarity of healthcare data towards their transformation to HL7 FHIR through ontology matching. Int J Med Inform. 2019;132:104002. doi: 10.1016/j.ijmedinf.2019.104002 31629311

[17] Xiao D, Song C, Nakamura N, Nakayama M. Development of an application concerning fast healthcare interoperability resources based on standardized structured medical information exchange version 2 data. Comput Methods Programs Biomed. 2021;208:106232. doi: 10.1016/j.cmpb.2021.106232 34174764

[18] Hartzema AG, Reich CG, Ryan PB, Stang PE, Madigan D, Welebob E, et al. Managing data quality for a drug safety surveillance system. Drug Saf. 2013;36 Suppl 1:S49-58. doi: 10.1007/s40264-013-0098-7 24166223

[19] Rahm E, Bernstein PA. A survey of approaches to automatic schema matching. The VLDB Journal. 2001;10(4):334–50. doi: 10.1007/s007780100057

[20] Banek M, Vrdoljak B, Tjoa AM, Skočir Z. Automating the schema matching process for heterogeneous data warehouses. In: Data warehousing and knowledge discovery: 9th international conference, DaWaK 2007, Berlin, Heidelberg: Springer; 2007. pp. 45–54.

[21] Bernstein PA, Madhavan J, Rahm E. Generic schema matching, ten years later. Proc VLDB Endow. 2011;4(11):695–701. doi: 10.14778/3402707.3402710

[22] Po L, Sorrentino S. Automatic generation of probabilistic relationships for improving schema matching. Inf Syst. 2011;36(2):192–208. doi: 10.1016/j.is.2010.09.004

[23] Rahm E. Towards large-scale schema and ontology matching. In: Schema matching and mapping. Springer; 2011. p. 3–27.

[24] Islam A, Inkpen D, Kiringa I. Applications of corpus-based semantic similarity and word segmentation to database schema matching. The VLDB Journal. 2007;17(5):1293–320. doi: 10.1007/s00778-007-0067-9

[25] Sutanta E, Wardoyo R, Mustofa K, Winarko E. Survey: models and prototypes of schema matching. Int J Electr Comput Eng. 2016;6(3).

[26] Newton JJ. Guide on data entity (Naming Conventions); 1987.

[27] ISO, Information technology — Metadata registries (MDR) — Part 5: Naming principles; 2015.

[28] Rachman MAF, Saptawati GAP. Database integration based on combination schema matching approach (case study: Multi-database of district health information system). In: 2017 2nd International conferences on Information Technology, Information Systems and Electrical Engineering (ICITISEE); 2017. pp. 430–5. 10.1109/icitisee.2017.8285544

[29] Alwan AA, Nordin A, Alzeber M, Abualkishik AZ. A survey of schema matching research using database schemas and instances. Int J Adv Comput Sci Appl. 2017;8(10).

[30] Algergawy A, Nayak R, Saake G. Element similarity measures in XML schema matching. Inf Sci. 2010;180(24):4975–98. doi: 10.1016/j.ins.2010.08.022

[31] OpenAi. OpenAi, ChatGPT; 2025.

[32] Google. An overview of the Gemini app. Gemini; 2025.

[33] DeepSeek. DeepSeek: into the unknown; 2025.

[34] Achiam J, Adler S, Agarwal S, Ahmad L, Akkaya I, Aleman FL, et al. Gpt-4 technical report. arXiv preprint. 2023. doi: 10.48550/arXiv.2303.08774

[35] Rahman A, Mahir SH, Tashrif MT, Karim MA, Aishi AA, Kundu D, et al. Comparative analysis based on DeepSeek, ChatGPT, and Google Gemini: features, techniques, performance, future prospects; 2025.

[36] DeepSeek AI. DeepSeek-V3 Technical Report; 2025.

[37] International HL. Common data models harmonization; 2021.

[38] Powers DM. Evaluation: from precision, recall and F-measure to ROC, informedness, markedness and correlation. arXiv preprint 2020:arXiv:2010.16061.

[39] Faria D, Santos E, Balasubramani BS, Silva MC, Couto FM, Pesquita C. Agreementmakerlight. Semant Web. 2025;16(2):SW-233304.

[40] Zhang J, Shin B, Choi JD, Ho JC. SMAT: an attention-based deep learning solution to the automation of schema matching. Cham: Springer International Publishing; 2021. 10.1007/978-3-030-82472-3_19 PMC8487677 34608464

[41] Sadruddin S, D’Souza J, Poupaki E, Watkins A, Babaei Giglou H, Rula A, et al. LLMs4SchemaDiscovery: a Human-in-the-Loop workflow for scientific schema mining with large language models. arXiv preprint. 2025.

